# PINK1 positively regulates IL-1β-mediated signaling through Tollip and IRAK1 modulation

**DOI:** 10.1186/1742-2094-9-271

**Published:** 2012-12-17

**Authors:** Hyun Jung Lee, Kwang Chul Chung

**Affiliations:** 1 Department of Systems Biology, College of Life Science and Biotechnology, Yonsei University, Yonsei-ro 50, Seodaemun-gu, Seoul, 120-749, Korea

**Keywords:** IL-1β, Inflammation, Parkinson disease, PTEN-induced putative kinase 1, Toll-interacting protein, IL-1 receptor-associated kinase 1

## Abstract

**Background:**

Parkinson disease (PD) is characterized by a slow, progressive degeneration of dopaminergic neurons in the substantianigra. The cause of neuronal loss in PD is not well understood, but several genetic loci, including PTEN-induced putative kinase 1 (PINK1), have been linked to early-onset autosomal recessive forms of familial PD. Neuroinflammation greatly contributes to PD neuronal degeneration and pathogenesis. IL-1 is one of the principal cytokines that regulates various immune and inflammatory responses via the activation of the transcription factors NF-κB and activating protein-1. Despite the close relationship between PD and neuroinflammation, the functional roles of PD-linked genes during inflammatory processes remain poorly understood.

**Methods:**

To explore the functional roles of PINK1 in response to IL-1β stimulation, HEK293 cells, mouse embryonic fibroblasts derived from PINK1-null (*PINK1*^*−/−*^) and control (*PINK1*^*+/+*^) mice, and 293 IL-1RI cells stably expressing type 1 IL-1 receptor were used. Immunoprecipitation and western blot analysis were performed to detect protein–protein interaction and protein ubiquitination. To confirm the effect of PINK1 on NF-κB activation, NF-κB-dependent firefly luciferase reporter assay was conducted.

**Results:**

PINK1 specifically binds two components of the IL-1-mediated signaling cascade, Toll-interacting protein (Tollip) and IL-1 receptor-associated kinase 1 (IRAK1). The association of PINK1 with Tollip, a negative regulator of IL-1β signaling, increases upon IL-1β stimulation, which then facilitates the dissociation of Tollip from IRAK1 as well as the assembly of the IRAK1–TNF receptor-associated factor 6 (TRAF6) complex. PINK1 also enhances Lys63-linked polyubiquitination of IRAK1, an essential modification of recruitment of NF-κB essential modulator and subsequent IκB kinase activation, and increases formation of the intermediate signalosome including IRAK1, TRAF6, and transforming growth factor-β activated kinase 1. Furthermore, PINK1 stimulates IL-1β-induced NF-κB activity via suppression of Tollip inhibitory action.

**Conclusions:**

These results suggest that PINK1 upregulates IL-1β-mediated signaling through the functional modulation of Tollip and IRAK1. These results further suggest that PINK1 stimulates the ubiquitination of proximal molecules and increases signalosome formation in the IL-1β-mediated signaling pathway. The present study therefore supports the idea of the close relationship between neuroinflammation and PD.

## Introduction

Parkinson disease (PD) is characterized by progressive dopaminergic neuron loss in the substantianigra pars compacta of the midbrain [1]. Although the etiology of PD remains poorly understood, several genetic loci have been implicated in the pathogenesis of familial PD. Among them, the PTEN-induced putative kinase 1 (PINK1) gene encodes a serine/threonine kinase that phosphorylates substrates such as TNF receptor-associated protein 1 and thus protects cells from death induced by various stress signals [2,3]. Neuroinflammatory processes, including microglial activation, astrogliosis, and lymphocytic infiltration, significantly contribute to neuronal degeneration by producing deleterious proinflammatory molecules [4].

Toll-like receptors (TLRs) are a family of evolutionarily conserved receptors that recognize pathogen-associated molecular patterns, causing an inflammatory response via induction of interleukins and other proinflammatory proteins [5]. Toll-interacting protein (Tollip) is an adaptor protein that acts as an inhibitory factor in TLR signaling cascades [6-8]. When activated by IL-1 or lipopolysaccharide stimulation, Tollip associates with the cytoplasmic TIR domains of IL-1 receptor (IL-1R), as well as TLR2 and TLR4 [6,7,9]. Tollip also interacts with IL-1 receptor-associated kinase 1 (IRAK1) and suppresses its kinase activity [7]. Therefore, in the absence of infection, Tollip probably maintains immune cells in a resting state and terminates IL-1R-induced and TLR-induced inflammatory pathways via suppression of IRAK1 activity [7,9]. In this regard, Tollip resembles IL-1R-associated kinase M, which also acts as a negative regulator in IL-1β signaling. IL-1R-associated kinase M associates with IRAK1 by blocking IL-1R-associated kinase 4 recruitment, and thereby inhibits IRAK1 phosphorylation and/or activation [10,11].

IRAK1 is an adaptor for the Toll/IL-1R receptor signaling complex. Upon IL-1 stimulation, formation of the heterodimeric receptor complex creates a scaffold for the association of MyD88 and Tollip [12-14]. IRAK1 is then recruited to the active receptor complex. In parallel, IL-1R-associated kinase 4 is recruited to the receptor complex and may phosphorylate IRAK1, thus initiating further autophosphorylation of IRAK1. Hyperphosphorylated IRAK1 dissociates from the receptor complex, presumably dimerizes, and binds to TNF receptor-associated factor 6 (TRAF6). IRAK1 binding to TRAF6 functions together with Ubc13/Uev1A to catalyze Lys63-polyubiquitination of IRAK1. Active IRAK1 subsequently causes the dimerization and polyubiquitination of TRAF6, which activates the downstream component, transforming growth factor-β activated kinase 1 (TAK1). TAK1 subsequently phosphorylates several regulatory kinases in different downstream signaling pathways, which ultimately leads to the production and release of multiple cytokines via NF-κB activation [15].

We previously revealed that PINK1 directly binds two components of IL-1β-mediated downstream signaling, TRAF6 and TAK1 [16]. In addition, PINK1 overexpression maintains the ubiquitination and phosphorylation of these two proteins in 293 IL-1RI cells, consequently resulting in more potent NF-κB activity [16]. Furthermore, silencing PINK1 expression by RNA interference or the overexpression of the kinase-defective PINK1 mutant suppresses TRAF6 and TAK1 activity and inhibits IL-1β-induced NF-κB activation. These results suggest that PINK1 stimulates the IL-1β response by positively regulating TRAF6 and TAK1. In the present study, we aimed to investigate whether PINK1 additionally affects other upstream molecules in the IL-1β signaling pathway as well as the formation of the IRAK1, TRAF6, and TAK1 intermediate signalosome complex. We demonstrate that PINK1 blocks the inhibitory action of Tollip upon IL-1β stimulation. In addition, PINK1 positively regulates IL-1β-mediated NF-κB activation via IRAK1 modulation. These findings provide a novel regulatory role for Tollip and IRAK1 activity during inflammatory signaling.

## Materials and methods

### Materials

DMEM, FBS, FCS, and the LipofectAMINE PLUS reagent were purchased from Invitrogen (Carlsbad, CA, USA). Protein A-Sepharose was obtained from GE Healthcare Biosciences (Piscataway, NJ, USA) and anti-PINK1 antibody was purchased from Abgent (San Diego, CA, USA). The HA, c-Myc, Tollip, IRAK1, TRAF6, TAK1, actin, and ubiquitin antibodies were purchased from Santa Cruz Biotechnology (Santa Cruz, CA, USA). Secondary goat anti-IgG and horseradish peroxidase-conjugated anti-rabbit and anti-mouse IgGs were purchased from Life Technologies (Grand Island, NY, USA). Flag antiserum and recombinant human IL-1β were obtained from Sigma (St Louis, MO, USA). Enhanced chemiluminescencereagent was purchased from Perkin-Elmer Life and Analytical Sciences (Waltham, MA, USA). The Lys63-specific anti-ubiquitin antibody was obtained from Millipore (Temecula, CA, USA). All other chemicals used in the study were analytical grade commercial products purchased from Sigma.

### DNA constructs

Plasmids encoding Flag-IRAK1, Flag-TAK1 and Flag-TRAF6 were kindly provided by G Takaesu (Keio University, Tokyo, Japan). The mammalian constructs encoding HA-tagged Tollip and Myc-tagged wild-type hPINK1 (pBOS-3X-myc-hPINK1-WT) were obtained from K Nakayama (Kyoto University, Kyoto, Japan) and J Chung (Seoul National University, Seoul, Korea), respectively. The plasmid encoding the Myc-tagged kinase-deficient hPINK1 mutant (multi-point mutations at K219A, D362A, and D384A, termed pBOS-3X-myc-hPINK1-KD) was generated using the QuikChangeXL site-directed mutagenesis kit (Stratagene, La Jolla, CA, USA), according to the manufacturer’s protocol. PCR was performed using the following primers (mutated codon is underlined in each primer): K219A, forward primer 5′-CCCTTGGCCATC**GCG**ATGATGTGGAAC-3′ and reverse primer 5′-GTTCCACATCAT**CGC**GATGGCCAAGGG-3′; D362A, forward primer 5′-ATCGCGCACAGA**GCC**CTGAAATCCGAC-3′ and reverse primer 5′-GTCGGATTTCAG**GGC**TCTGTGCGCGAT-3′; D384A, forward primer 5′-CTGGTGATCGCA**GCT**TTTGGCTGCTGC-3′ and reverse primer 5′-GCAGCAGCCAAA**AGC**TGCGATCACCAG-3′. The plasmid encoding V5-TRAF6 was generated by subcloning TRAF6 from pCMV-Flag-TRAF6 into the pcDNA3.1/V5-His vector using *EcoR*I and *Xho*I sites.

### Cell culture and DNA transfection

Mouse embryonic fibroblasts (MEFs) derived from PINK1-null (*PINK1*^*−/−*^) and control (*PINK1*^*+/+*^) mice were provided by J Shen (Harvard Medical School, Boston, MA, USA), and 293 IL-1RI cells stably expressing type 1 IL-1R were a kind gift from G Takaesu (Keio University). Human embryonic kidney cells (HEK293), *PINK1*^*−/−*^, and *PINK1*^*+/+*^ MEFs were cultured in DMEM containing 10% FBS, 100 units/ml penicillin, and 100 μg/ml streptomycin at 37°C in 5% CO_2_. The 293 IL-1RI cells were cultured in DMEM containing 10% FCS, 100 units/ml penicillin, and 100 μg/ml streptomycin at 37°C in 5% CO_2_. DNA transfection was performed using the LipofectAMINE PLUS reagent (Invitrogen) according to the manufacturer’s protocol. The total amount of DNA transfected for each condition was adjusted using parental empty vector DNA.

### Immunoprecipitation and immunoblot assay

Cells were rinsed twice with ice-cold PBS, harvested in 1% Nonidet P40 lysis buffer (50 mM Tris, pH 7.5, 1% Nonidet P40, 150 mM NaCl, 10% glycerol, 1 mM Na_3_VO_4_, 1 μg/ml leupeptin, 1 μg/ml aprotinin, 1 mM ethylene glycol tetraacetic acid, 1 mM ethylene diamine tetraacetic acid, 10 mM NaF, and 0.2 mM phenylmethylsulfonyl fluoride), and briefly sonicated. Lysates were collected by centrifugation at 13,000 × *g* for 20 minutes at 4°C. For immunoprecipitation, 1 μg appropriate antibody was incubated overnight at 4°C with 0.5 to 1 mg cell extracts prepared in cell lysis buffer. Thirty microliters of a 1:1 suspension of protein A-sepharose beads were added and incubated for 2 hours at 4°C with gentle rotation. Beads were pelleted by centrifugation at 10,000 × *g* for 30 seconds at 4°C, and washed three times with 1% Nonidet P40 lysis buffer. Immunocomplexes were dissociated by boiling in SDS-PAGE sample buffer, separated by SDS-PAGE, and transferred to a nitrocellulose membrane. Membranes were blocked for 1 hour at room temperature in TBST buffer (20 mM Tris, pH 7.5, 137 mM NaCl, and 0.1% Tween 20) containing 5% nonfat dry milk, followed by overnight incubation at 4°C in TBST buffer containing 3% nonfat dry milk and the appropriate primary antibody. Membranes were washed three times in TBST and then incubated for 1 hour at room temperature with the secondary IgG-coupled horseradish peroxidase antibody. The membranes were washed three times with TBST, and the signals were visualized with enhanced chemiluminescence reagent.

### Immunocytochemistry

After transfection, cells were washed twice with ice-cold PBS (pH 7.4), fixed with 3.7% formaldehyde in PBS for 15 minutes, permeabilized with 0.2% Triton X-100 for 20 minutes, blocked with 1% bovine serum albumin for 30 minutes, and incubated overnight at 4°C with the primary antibody. After washing with PBS, cells were incubated for 2 hours with a FITC-conjugated or TRITC-conjugated secondary antibody. Where specified, samples were stained with 4′,6′-diamidino-2-phenylindole using the SlowFade Antifade kit (Invitrogen). Fixed cells were visualized using a LSM-510 META confocal microscope (Carl Zeiss, Gottingen, Germany).

### Luciferase reporter assay

*PINK1*^*+/+*^ and *PINK1*^*−/−*^ MEF cells were grown for 24 hours in six-well plates at a density of 3 × 10^5^ cells/well. NF-κB-dependent firefly luciferase reporter plasmids and the renilla luciferase plasmid were co-transfected into the cells. Forty-eight hours after transfection, the cells were harvested in passive lysis buffer (Promega, Madison, WI, USA) and luciferase assays were performed using the Dual-Luciferase Reporter Assay System (Promega). Relative luciferase activity was calculated by dividing firefly luciferase activity by renilla luciferase activity. Data represent three independent experiments performed in triplicate.

### Statistical analysis

Statistical differences were determined using one-way analysis of variance with the Tukey*post hoc* test. All values were expressed as mean ± standard deviation.

## Results

### PINK1 physically interacts with Tollip and IRAK1 in mammalian cells

**Figure 1:**
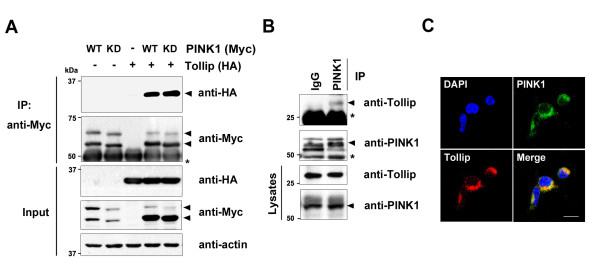
**PINK1 binds Tollip in HEK293 cells.** (**A**) HEK293 cells were transfected for 48 hours with Myc-tagged PTEN-induced putative kinase 1 (PINK1) and/or HA-tagged Toll-interacting protein (Tollip). Immunoprecipitation (IP) of cell lysates was performed with the c-Myc antibody, followed by immunoblotting with the HA antibody, as indicated. Expression of the transiently transfected proteins was identified by immunoblotting with the indicated antibodies. *IgG heavy chain. Actin was used as a loading control. (**B**) HEK293 cell lysates were immunoprecipitated with the PINK1 antibody, followed by immunoblotting with the Tollip antibody. As a control, cell lysates were immunoprecipitated with preimmune IgG. PINK1 and Tollip expression in cell extracts was determined by immunoblotting with each antibody. (**C**) HEK293 cells were fixed, permeabilized, and incubated for 24 hours with the PINK1or Tollip antibody. Cells were stained with TRITC-conjugated or FITC-conjugated secondary antibodies and 4′,6′-diamidino-2-phenylindole (DAPI). Immunostained cells were examined using confocal microscopy. Scale bar = 20 μm.

To examine the role of PINK1 during inflammation, we performed a yeast two-hybrid assay with full-length PINK1 as bait. We screened 5 × 10^6^ human fetal brain cDNA library clones and identified a number of unknown, as well as previously reported, PINK1-binding partners, including parkin [17], TRAF6 [16], Tollip, and IRAK1 (data not shown). To determine the role of PINK1 in IL-1β-induced inflammatory signaling, we first determined whether PINK1 binds Tollip in mammalian cells using co-immunoprecipitation assays. To examine whether PINK1 can bind Tollip under normal growth conditions, HEK293 cells were co-transfected with HA-tagged Tollip and either Myc-tagged wild-type hPINK1 or its kinase-deficient mutant (hPINK1-KD) alone or in combination. Cell lysates were immunoprecipitated using anti-c-Myc IgG, followed by immunoblot analyses with the HA antibody. Consistent with the previous reports [18,19], ectopic expression of PINK1 generated two bands, as shown in western blot analyses (Figure 1A). The upper band represents full-length PINK1, whereas the lower band represents the processed isoform of PINK1 from mitochondria to cytosol. Compared with PINK1 mutant, the wild-type band appeared to be upper-shifted, confirming that wild-type PINK1, but not its kinase-deficient mutant, is a target of autophosphorylation. In addition, transiently transfected wild-type PINK1 selectively bound Tollip in HEK293 cells (Figure 1A). Moreover, the kinase-deficient PINK1 mutant still binds Tollip, suggesting that PINK1 kinase activity is not necessary for Tollip binding (Figure 1A). To determine whether endogenous PINK1 binds endogenous Tollip, HEK293 cell lysates were immunoprecipitated with anti-PINK1 IgG, followed by immunoblot analyses with the Tollip antibody. As shown in Figure 1B, endogenous PINK1 interacted with endogenous Tollip, whereas no obvious interaction was observed in control immunocomplex samples prepared with preimmune IgG (Figure 1B). These data suggest that the PINK1/Tollip interaction is not an artifact of DNA transfection but a specific interaction in mammalian systems. Moreover, immunocytochemical analyses revealed that endogenous PINK1 co-localizes with endogenous Tollip, predominantly in the cytoplasmic region of HEK293 cells (Figure 1C). Together, these results suggest that PINK1 specifically binds Tollip in mammalian cells.

**Figure 2:**
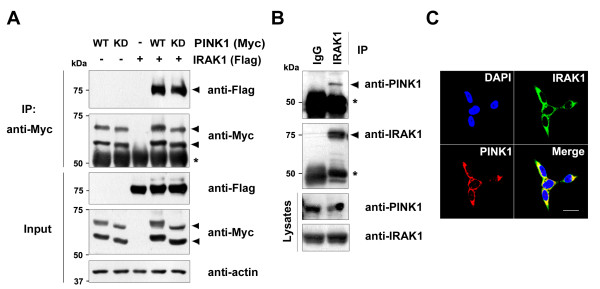
**PINK1 physically interacts with IRAK1 in HEK293 cells.** (**A**) HEK293 cells were transfected for 48 hours with Myc-PINK1 and/or Flag-IRAK1. Cell lysates were immunoprecipitated with the c-Myc antibody, followed by immunoblotting with the Flag antibody. Expression of the transiently transfected proteins was identified by immunoblotting with the indicated antibodies. *IgG heavy chains. Actin was used as a loading control. (**B**) HEK293 cell lysates were immunoprecipitated with the IRAK1 antibody, followed by immunoblotting with the PINK1 antibody. As a control, cell lysates were immunoprecipitated with preimmune IgG. (**C**) HEK293 cells were fixed, permeabilized, and incubated for 24 hours with PINK1 or IRAK1 antibodies. Cells were stained with TRITC-conjugated or FITC-conjugated secondary antibodies and 4′,6′-diamidino-2-phenylindole (DAPI). Immunostained cells were examined using confocal microscopy. Scale bar = 20 μm. IRAK1, IL-1 receptor-associated kinase 1; PINK1, PTEN-induced putative kinase 1.

We next investigated whether PINK1 can also bind IRAK1 in mammalian cells. HEK293 cells were transiently transfected with Flag-tagged IRAK1 plus either Myc-tagged wild-type PINK1 (hPINK1-WT) or the kinase-defective mutant (hPINK1-KD). Anti-Flag immunocomplexes from these cells were subjected to western blot analyses using the c-Myc antibody. As shown in Figure 2A, wild-type and kinase-deficient PINK1 both bind IRAK1 in HEK293 cells. In addition, endogenous PINK1 directly associates with endogenous IRAK1 (Figure 2B). Finally, immunocytochemical analyses showed that endogenous PINK1 and endogenous IRAK1 co-localize in the cytoplasm of HEK293 cells (Figure 2C). These experiments thus suggest that PINK1 specifically binds Tollip and IRAK1 in mammalian cells.

### PINK1 facilitates the dissociation of Tollip–IRAK1 complex

**Figure 3:**
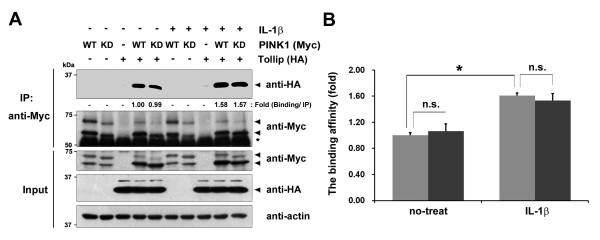
**Association between PINK1 and Tollip increases upon IL-1β stimulation.** (**A**),(**B**) 293 IL-1RI cells were transfected for 42 hours with Myc-hPINK1-WT, Myc-hPINK1-KD, or HA-Tollip, alone or in combination, and treated with 10 ng/ml IL-1β for 15 minutes. Cell lysates were subjected to immunoprecipitation (IP) with anti-Myc antibody, followed by immunoblotting with anti-HA antibody. Total cell lysates were also analyzed with anti-Myc and anti-HA antibodies. *IgG heavy chains. Actin served as a loading control. (**A**) The binding intensity ((Tollip bound/Tollip input)/PINK1 IP) was quantified and normalized to the intensity of IP using Image J software [22]. (**B**) *P<0.05; n.s., not significant.PINK1, PTEN-induced putative kinase 1; Tollip, Toll-interacting protein.

Based on reports that Tollip negatively regulates the IL-1β and TNFα signaling pathways [20], we further explored the functional consequence of PINK1 on Tollip activity, focusing on the regulation of IL-1β-mediated inflammatory signaling. First, we investigated whether IL-1β treatment changes the PINK1/Tollip interaction and whether their association is affected by PINK1 kinase activity. For this purpose, we utilized 293 IL-1RI cells stably expressing type I IL-1β receptor [21], which consequently show much higher response to IL-1β stimulation. After co-transfection with HA-Tollip plus either Myc-hPINK1-WT or Myc-hPINK1-KD, 293 IL-1RI cells were treated with IL-1β. Immunoblot analyses of the anti-Myc immunocomplexes with HA antibodies revealed that IL-1β stimulation enhances the association between PINK1 and Tollip by ~60% (Figure 3A, B). However, the increased Tollip binding was not remarkably changed in the presence of the kinase-defective PINK1 (Figure 3A, B).

**Figure 4:**
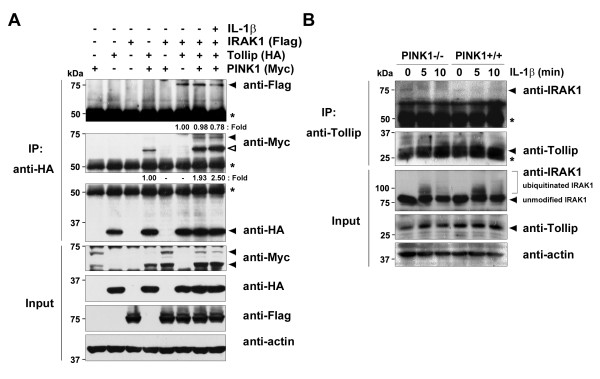
**PINK1 facilitates IRAK1 dissociation from Tollip after IL-1β stimulation.** (**A**) 293 IL-1RI cells were transfected for 42 hours with Myc-PINK1, HA-Tollip, or Flag-IRAK1, alone or in combination, and treated for 15 minutes with 10 ng/ml IL-1β. Cell lysates were immunoprecipitated with the HA antibody, followed by immunoblot analyses with the Flag or c-Myc antibodies. The relative binding affinities were quantified – (IRAK1 bound/IRAK1 input)/Tollip IP or (PINK1 bound/PINK1 input)/Tollip IP – and denoted below the upper panel. Open arrow, increased binding of PINK1 to Tollip. Actin used as a loading control. (**B**) *PINK1*^*−/−*^ or *PINK1*^*+/+*^mouse embryonic fibroblasts were treated with 50 ng/ml IL-1β for the indicated times. Cell lysates were immunoprecipitated with the Tollip antibody, and immunoblotted with the IRAK1 antibody. The relative binding affinities were quantified and denoted below the upper panel. *IgG heavy chains. Actin served as a loading control. IRAK1, IL-1 receptor-associated kinase 1; PINK1, PTEN-induced putative kinase 1; Tollip, Toll-interacting protein.

Although Tollip forms a binary complex with IRAK1 in the absence of ligand, this complex is recruited to IL-1R upon IL-1β stimulation [6]. IRAK1 is phosphorylated, leading to its rapid dissociation from Tollip, and causing IRAK1-induced TRAF6 activation [23]. Based on these reports, we investigated whether PINK1–Tollip binding affects the sequential protein interaction between Tollip and IRAK1. 293 IL-1RI cells were transfected with Myc-hPINK1, HA-Tollip, or Flag-IRAK1 alone or in combination and co-immunoprecipitation assays were performed. As expected, Tollip interacts strongly with IRAK1 regardless of PINK1 expression. Interestingly, the addition of PINK1 decreases Tollip binding to IRAK1 under IL-1β stimulation (Figure 4A). These results suggest that the IL-1β stimulation-mediated increased binding of PINK1 to Tollip (Figure 4A) reduces the affinity between Tollip and IRAK1, which subsequently dissociates Tollip from IRAK1.

To further examine whether PINK1 diminishes the binding of endogenous Tollip and IRAK1, we compared the binding pattern and levels in response to IL-1β stimulation in *PINK1*^*−/−*^ and *PINK1*^*+/+*^ MEFs. After MEFs were treated with IL-1β, cell lysates were immunoprecipitated with the Tollip antibody. Immunoblotting the complex with the IRAK1 antibody revealed that the endogenous association between IRAK1 and Tollip occurs more strongly in *PINK1*^*−/−*^ MEFs in the absence of IL-1β stimulation, whereas it was very weak in *PINK1*^*+/+*^ MEFs (Figure 4B). In addition, the endogenous Tollip/IRAK1 interaction occurs until 10 minutes after IL-1β stimulation in *PINK1*^*−/−*^ MEFs (Figure 4B). Together, these results suggest that PINK1 facilitates Tollip dissociation from IRAK1, which may consequently promote IL-1β-induced downstream signaling.

### PINK1 facilitates Lys63-linked polyubiquitination of IRAK1

**Figure 5:**
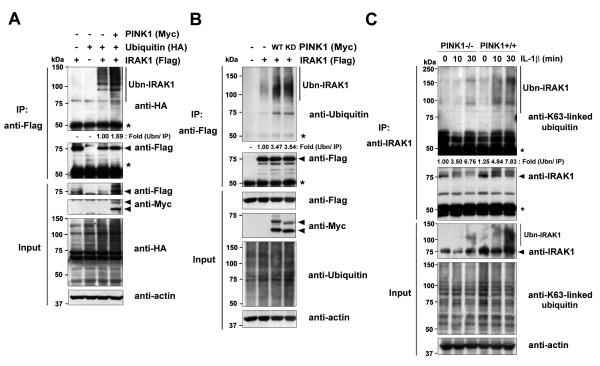
**PINK1 increases Lys63-dependent polyubiquitination of IRAK1.** (**A**) HEK293 cells were transfected for 48 hours with HA-ubiquitin, Flag-IRAK1, or Myc-PINK1, alone or in combination. Cell lysates were immunoprecipitated with the Flag antibody, followed by immunoblotting with the HA antibody. Expression of transiently transfected proteins was confirmed by immunoblotting with the HA, Flag, or c-Myc antibodies. *IgG heavy chains.Actin served as a loading control. (**B**) 293 IL-1RI cells were transfected for 42 hours with Flag-IRAK1 alone or together with either Myc-tagged wild-type PINK1 (WT) or its kinase-defective mutant (KD), and treated for 15 minutes with 10 ng/ml IL-1β. Cell lysates were immunoprecipitated with the Flag antibody, followed by immunoblotting with the ubiquitin antibody. (**C**) *PINK1*^*−/−*^ and *PINK1*^*+/+*^mouse embryonic fibroblasts were treated with 50 ng/ml IL-1β for the indicated times. Cell lysates were immunoprecipitated with the IRAK1 antibody, and immunoblotted with the Lys63-specific ubiquitin antibody. The relative polyubiquitinated IRAK1 levels were quantified and denoted below the upper panel:(A), (B) (ubiquitinated IRAK1/IRAK1 input)/IRAK1 IP, or (C) (endogenous ubiquitinated IRAK1/endogenous IRAK1)/endogenous IRAK1 IP.IRAK1, IL-1 receptor-associated kinase 1; PINK1, PTEN-induced putative kinase 1.

Recent studies reveal that IRAK1 undergoes Lys63-linked ubiquitination following IL-1 stimulation. This modification is essential for the recruitment of NF-κB essential modulator and subsequent IκB kinase (IKK) activation [24,25]. Given the importance of IRAK1 ubiquitination in IL-1 signaling, we determined whether PINK1 affects IRAK1 ubiquitination upon IL-1β stimulation by conducting *in vivo*ubiquitination assays. The 293 IL-1RI cells were co-transfected with Flag-IRAK1, HA-ubiquitin, or Myc-hPINK1, alone or in combination, and stimulated with IL-1β. Immunoblot analyses of the Flag immunocomplexes with the HA antibody demonstrate that PINK1 overexpression increases IRAK1 polyubiquitination (Figure 5A, lane 4) compared with cells expressing IRAK1 and ubiquitin alone (Figure 5A, lane 3).

Next, we assessed whether IRAK1 polyubiquitination depends on PINK1 kinase activity. The 293 IL-1RI cells were transfected with either Myc-hPINK1-WT or Myc-hPINK1-KD, along with Flag-tagged IRAK1, and then treated with IL-1β. Immunoblot analyses of the Flag immunocomplexes with the ubiquitin antibody revealed that wild-type PINK1 increases IRAK1 polyubiquitination by 3.5-fold (Figure 5B), whereas it is not significantly different in the presence of Myc-hPINK1-KD. These results suggest that PINK1 positively regulates IRAK1 polyubiquitination independent of its kinase activity.

We next addressed whether PINK1 facilitates Lys63-linked polyubiquitin conjugation to endogenous IRAK1 in response to IL-1β because this is required for IKK activation. *PINK1*^*−/−*^ and *PINK1*^*+/+*^ MEFs were stimulated with IL-1β and cell lysates were immunoprecipitated with the IRAK1 antibody (Figure 5C). Immunoblot analyses with the Lys63-specific ubiquitin antibody showed that Lys63-dependent polyubiquitination of IRAK1 in *PINK1*^*−/−*^ MEFs only slightly increases after 30 minutes of IL-1β stimulation. In contrast, IRAK1 polyubiquitinationin *PINK1*^*+/+*^ MEFs significantly increased after 10 minutes of IL-1β treatment, reaching its maximum after 30 minutes. These results indicate that PINK1 promotes Lys63-specific polyubiquitination of IRAK1 in mammalian cells.

### PINK1 promotes assembly of IRAK1–TRAF6 complex

**Figure 6:**
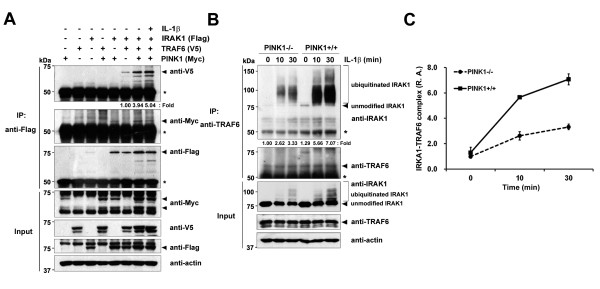
**PINK1 facilitates assembly of the IRAK1–TRAF6 complex.** (**A**) 293 IL-1RI cells were transfected for 48 hours with Flag-IRAK1, V5-TRAF6, or Myc-PINK1, alone or in combination, and treated for 15 minuteswith 10 ng/ml IL-1β. Cells were immunoprecipitated with the Flag antibody, followed by immunoblotting with the V5 or c-Myc antibodies. (**B**) *PINK1*^*−/−*^ or *PINK1*^*+/+*^mouse embryonic fibroblasts were treated with 50 μM MG132 for 30 minutes followed by 50 ng/ml IL-1β for the indicated times. Cell lysates were immunoprecipitated with the TRAF6 antibody, and immunoblotted with the IRAK1 antibody. The relative binding affinities were quantified and denoted below the upper panel: (A)(TRAF6 bound/TRAF6 input)/IRAK1 IP,or(B) (endogenous IRAK1 bound/endogenous IRAK1)/endogenous TRAF6 IP. *IgG heavy chains. Actin was used as a loading control. The binding intensity in (B) was quantified and error bars indicate ± standard deviation in triplicate experiments (**C**). IRAK1, IL-1 receptor-associated kinase 1; PINK1, PTEN-induced putative kinase 1;R.A., relative amounts; TRAF6, TNF receptor-associated factor 6.

Upon dissociation from IL-1R, IRAK1 and TRAF6 form another multi-protein complex at the plasma membrane consisting of TAK1, TAB1, and TAB2 [26-28]. These findings led us to investigate whether PINK1 affects formation of the IRAK1-TRAF6 complex after IL-1β stimulation. 293 IL-1RI cells were transiently transfected with Flag-IRAK1 and V5-TRAF6, alone or together with Myc-hPINK1, and treated with IL-1β. Immunoblot analyses of the Flag immunocomplexes with the V5 antibody revealed that IRAK1/TRAF6 binding occurs weakly in the absence of PINK1 (Figure 6A). However, PINK1 overexpression enhances their interaction under normal growth conditions. Moreover, PINK1 remarkably increased the IRAK1/TRAF6 interaction upon IL-1β stimulation (Figure 6A), suggesting that PINK1 facilitates IRAK1 dissociation from Tollip, which, in turn, promotes IRAK1 binding to TRAF6.

To examine whether PINK1 promotes the association of endogenous IRAK1 and TRAF6, we compared levels of the IRAK1–TRAF6 complex in response to IL-1β stimulation in *PINK1*^*−/−*^ and *PINK1*^*+/+*^ MEFs. The MEFs were treated sequentially with the proteasomal inhibitor MG132 and IL-1β, and cell lysates were immunoprecipitated with the TRAF6 antibody. Immunoblotting with the IRAK1 antibody confirmed that PINK1 significantly increases the IRAK1–TRAF6 complex (Figure 6B).

### PINK1 enhances formation of the intermediate signalosome, including IRAK1, TRAF6 and TAK1, upon IL-1β stimulation

**Figure 7:**
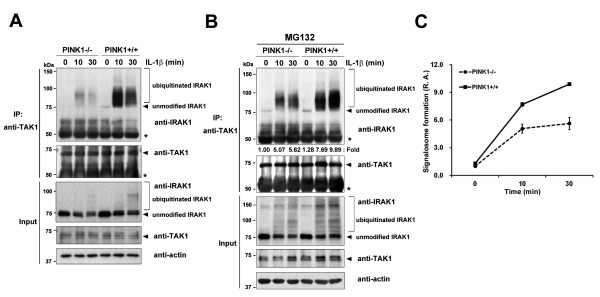
**PINK1 enhances formation of the intermediate signalosome including IRAK1, TRAF6, and TAK1.** (**A**),(**B**) *PINK1*^*−/−*^ or *PINK1*^*+/+*^ mouse embryo fibroblasts were pretreated with 50 μM MG132 for 30 minutes(B) and stimulated with 50 ng/ml IL-1β for the indicated times. Cell lysates were immunoprecipitated with the transforming growth factor-β activated kinase 1 (TAK1) antibody, followed by immunoblotting with the IL-1 receptor-associated kinase 1 (IRAK1) antibody. The relative binding affinities ((endogenous IRAK1 bound/endogenous IRAK1)/endogenous TAK1 IP) of samples were quantified and denoted below the upper panel. The binding intensity in (B) was quantified and error bars indicate ± standard deviation in triplicate experiments (**C**). PINK1, PTEN-induced putative kinase 1; R.A., relative amounts.

Lys63-linked polyubiquitination of IRAK1 and TRAF6 facilitates recruitment of TAK1 and IKK into the complex [29]. In addition, IL-1β treatment recruits endogenous TAK1 to the TRAF6 complex, a crucial step for activation of TAK1 catalytic activity, and subsequently triggers NF-κB and mitogen-activated protein kinase. Based on these reports, we investigated whether, upon IL-1β stimulation, PINK1 influences formation of the intermediate signalosome containing IRAK1, TRAF6, and TAK1. We used *PINK1*^*−/−*^ or *PINK1*^*+/+*^ MEFs to determine the effect of PINK1 on formation of the endogenous signalosome. *PINK1*^*−/−*^ and *PINK1*^*+/+*^ MEFs were stimulated with IL-1β for the indicated times (Figure 7A), and cell lysates were immunoprecipitated with the TAK1 antibody. Immunoblot analyses with the IRAK1 antibody revealed that PINK1 facilitates formation of the TRAF6-mediated IRAK1–TAK1 complex (Figure 7A). In addition, western blotting with the IRAK1 antibody showed that, in response to IL-1β, there is a time-dependent increase in the upper IRAK1 bands in *PINK1*^*+/+*^ MEFs. These slower migrating IRAK1 bands are presumed to result from hyperphosphorylation and ubiquitin modification [30,31]. To obtain enhanced levels of the PINK1-induced intermediate signalosome complex, cells were pretreated with MG132 before IL-1β stimulation (Figure 7B). Suppression of intracellular proteasomal activity led to an accumulation of ubiquitinated IRAK1 as well as unmodified IRAK1. Moreover, both modified and unmodified IRAK1 levels were enhanced in *PINK1*^*+/+*^MEFs, causing a robust recruitment of the intermediate signalosome. Taken together, these results indicate that PINK1 promotes assembly of the IRAK1–TRAF6–TAK1 complex, a crucial step for IKK and NF-κB activation.

### PINK1 potentiates NF-κB activity via Tollip suppression in response to IL-1β

**Figure 8:**
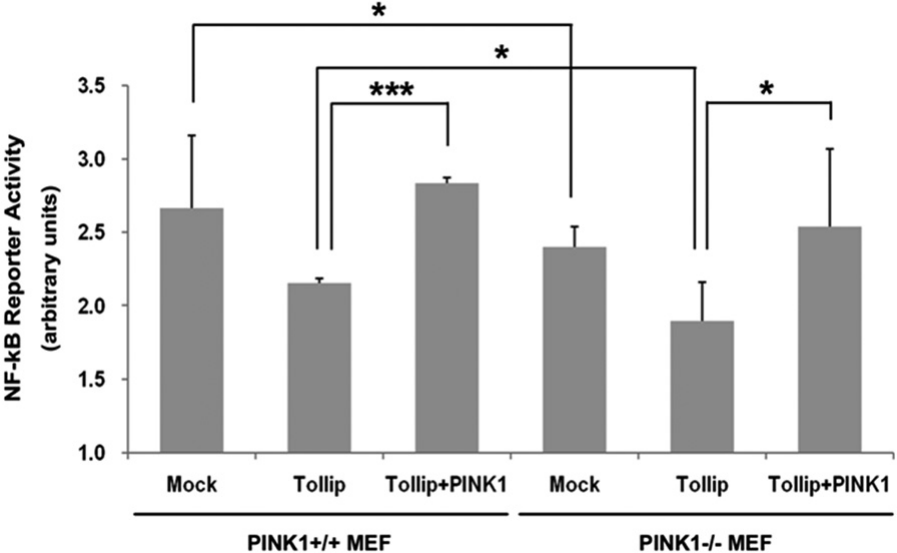
**PINK1 potentiates NF-κB activity via Tollip suppression in response to IL-1β.***PINK1*^*−/−*^ and *PINK1*^*+/+*^ mouse embryo fibroblasts were transfected for 36 hours with the NF-κB-responsive luciferase reporter and control renilla luciferase reporter alone or together with HA-Tollip and Myc-hPINK1-WT, as indicated. After cells were treated for 12 hourswith 50 ng/ml IL-1β, relative luciferase activity was measured and normalized to renilla activity. Error bars indicate ± standard deviation in triplicate experiments.***P<0.001. PINK1, PTEN-induced putative kinase 1.

To confirm that PINK1 positively regulates IL-1β-induced proximal signaling, we examined the effect of PINK1 on NF-κB activation. *PINK1*^*−/−*^ or *PINK1*^*+/+*^ MEFs were transfected with Tollip or PINK1, and stimulated with IL-1β, and the lysates were evaluated for NF-κB transactivation activity. As predicted, ectopic Tollip expression inhibits NF-κB activity, compared with mock-transfected cells. In addition, PINK1 overexpression into *PINK1*^*+/+*^ MEFs abrogated the inhibitory action of Tollip and increased NF-κB activity (Figure 8). Moreover, compared with the mock-transfected *PINK1*^*−/−*^ MEFs, Tollip expression significantly inhibited NF-κB activity but the ectopic expression of PINK1 elevated NF-κB activity. Taken together, these results indicate that PINK1 potentiates IL-1β-induced NF-κB activity through suppression of Tollip activity.

## Discussion

Neuroinflammation is thought to contribute to the cascade of events leading to neuronal degeneration in many neurodegenerative diseases, including PD. These events comprise microglial activation, astrogliosis, and lymphocytic infiltration. Despite intensive research, the relationship between PD-linked genes and neuroinflammatory signaling cascades is poorly understood. Recently, we reported that PINK1 participates in the IL-1β-mediated inflammatory signaling pathway through upregulation of its downstream molecules, TRAF6 and TAK1 [16]. PINK1 enhances TRAF6 autodimerization and autoubiquitination, which are essential for TRAF6 E3 activity and TAK1 polyubiquitination. Furthermore, PINK1 increases Lys63-dependent polyubiquitination of TAK1 and also directly phosphorylates TAK1 [16]. As a result, PINK1 potentiates NF-κB activity upon IL-1β stimulation. These novel findings suggest that a PD-linked gene may affect neuroinflammation by directly associating with and positively regulating key enzymes in IL-1β signaling. In the present study, we further examined whether PINK1 modulates the initial steps of IL-1β-induced inflammatory signaling, focusing on Tollip and IRAK1.

We first demonstrated that PINK1 specifically binds Tollip. The PINK1/Tollip association increased upon IL-1β stimulation, which facilitated Tollip dissociation from IRAK1 as well as assembly of the IRAK1–TRAF6 complex. In addition to functioning as a negative IRAK1 regulator, Tollip modulates IL-1RI signaling cascade in many ways, including sorting IL-1RI at late endosomes via formation with Tom1 and clathrin [32-34] and serving as a component of the IL-1RI sumoylation machinery [35]. Regarding Tollip-mediated IRAK1inhibition, the region near the Tollip UBA domain binds unphosphorylated IRAK1 in normal growth conditions and can also be phosphorylated by IRAK1 [7]. Therefore, once activated on the receptor, IRAK1 phosphorylates Tollip, which may dissociate Tollip from IRAK1 and the receptor complex. Taken together, our previous and present findings demonstrate that PINK1 effectively modulates several sequential steps of IL-1β signaling through direct binding to multiple upstream and downstream proteins in the IL-1β-mediated signaling pathway and modulating their interactions. In addition, PINK1 positively influences IL-1β-induced inflammation by direct phosphorylation of TAK1 [16].

Similar to PINK1, we also reported that the RCAN1-1S protein, a negative regulator of the phosphatase calcineurin, binds Tollip and positively modulates IL-1R-mediated signaling pathways by regulating Tollip–IRAK1–TRAF6 complex formation [36]. Unlike PINK1, RCAN1-1S interacts with Tollip and TRAF6 but not IRAK1, which leads to IRAK1 and TRAF6 dissociation from Tollip. RCAN1-1S also stimulates IL-1R-mediated signaling pathways, including TAK1 activation, NF-κB transactivation, and IL-8 production, which are all downstream of IL-1R activation [36].

Little is known about the putative PINK1-binding targets and/or substrates largely because of technical difficulties testing PINK1 kinase activity *in vivo*. However, several mitochondrial binding proteins have been identified, including parkin, Omi/HtrA2, and Rictor [37-39]. Additionally TNF receptor-associated protein 1 is a PINK1 substrate and is a key component mediating the cytoprotective actions of PINK1 [40]. Very recently, PINK1 was shown to phosphorylate Miro, a component of the primary motor/adaptor complex that anchors kinesin to the mitochondrial surface [41]. Moreover, we recently demonstrated that PINK1 directly phosphorylates TAK1, whereas it binds to TRAF6 and TAK1 [16]. In the present work, we found that PINK1 binds to Tollip and IRAK1, but their functional alterations are not via direct phosphorylation because wild-type and kinase-defective PINK1 influence them to a similar extent. PINK1’s mechanism of action is analogous to the well-characterized extracellular signal-regulated kinase 2 (ERK2) that activates poly(ADP-ribose) polymerase 1, topoisomerase II, and MKP-1 by direct protein interaction, independent of its kinase function [42]. In addition, ERK1 and ERK2 regulate cell cycle entry by disrupting the retinoblastoma pocket protein and laminA interaction in a kinase-independent fashion [42].

Increasing evidence indicates that PINK1 is localized in both the mitochondria and cytosol [18,19,43,44]. These reports suggest PINK1 has a dual localization and possibly two different functions, depending on its cellular localization [44,45]. In the cytoplasm, PINK1 forms a complex with parkin and DJ-1, which promotes the ubiquitination and degradation of parkin substrates in neuroblastoma cells [46]. Furthermore, PINK1 exerts its cytoprotective function in the mitochondria as well as in the cytoplasm through activation of mTORC2 and Akt [40]. In addition, our recent findings [16] and the current study suggest that an additional role for PINK1 in the cytoplasm is regulation of IL-1β-mediated upstream signaling via modulation of multiple targets.

We additionally demonstrated that PINK1 activates IRAK1 and facilitates assembly of the IRAK1–TRAF6 complex upon IL-1β stimulation. IRAK1 appears to play a crucial role in facilitating the multi-protein signaling complex or signalosome. IRAK1 links the active receptor complex to the central adaptor and co-activator protein TRAF6. Thus, IRAK1 may be a switch molecule that turns on signalosome formation (mediated through TRAF6 dimerization and TAB2 recruitment) [47]. Specifically, activation of the TLR/IL-1R signaling cascade induces rapid IRAK1 autophosphorylation, transient IRAK1 activation with the appearance of higher molecular weight forms, and the loss of IRAK1 protein [30]. Decreased IRAK1 levels correlate with the covalent attachment of Lys48-linked polyubiquitin and the targeting of IRAK1 for proteasomal degradation. TRAF6 and Pellino participate in Lys63-linked polyubiquitination of IRAK1 [24,47,49], which consequently recruits NF-κB essential modulator for subsequent NF-κB activation [24,25]. In fact, Tollip overexpression dramatically inhibits IRAK1 kinase activity [7] and suppresses NF-κB activation [6,7,9].

We observed the polyubiquitination of endogenous IRAK1 increases in *PINK1*^*+/+*^ MEFs, compared with *PINK1*^*−/−*^ cells. Furthermore, the IRAK1 protein level is enhanced in *PINK1*^*+/+*^ MEFs. These data suggest that PINK1 stimulates Lys63-linked polyubiquitination of IRAK1, but delays Lys48-linked polyubiquitination of IRAK1 and proteasomal degradation. In this manner, upon IL-1β stimulation, PINK1 may facilitate signalosome formation containing IRAK1, TRAF6, and TAK1. These data were further supported by the finding that Tollip overexpression impairs NF-κB activation, which is rescued by co-expression of wild-type PINK1.

Several molecules mediate IRAK1 ubiquitination, including TRAF6 and Pellino. For example, TRAF6 functions as an E3 ubiquitin ligase, together with Ubc13/Uev1A, which conjugates Lys63-linked polyubiquitin chains to itself as well as IRAK1 and subsequently recruits and activates IKK [24]. The three mammalian Pellino proteins possess C-terminal RING-like domains that catalyze IRAK polyubiquitination. Pellino proteins are phosphorylated by IRAK1 and IL-1R-associated kinase 4, which enhances Pellino E3 ligase activity [48]. Furthermore, IRAK1 kinase activity promotes the Pellino protein degradation and this might be an important regulatory mechanism that controls TLR signaling. A recent report proposed that Pellino3 negatively regulates TAK1-mediated activation of NF-κB, with inhibitory effects dependent on Pellino3-mediated Lys63-linked polyubiquitination of IRAK1 [49]. The authors propose that, upon IL-1β stimulation, Pellino3-mediated Lys63-linked IRAK polyubiquitination competes with Lys48-linked IRAK polyubiquitination for the same ubiquitination site, Lys134 of IRAK. Pellino3 therefore blocks IRAK degradation and thus inhibits IL-1-induced NF-κB activation.

PINK1 also stimulates signalosome complex formation, involving IRAK1, TRAF6, and TAK1. In addition, IL-1R-associated kinase 4 binds and phosphorylates IRAK1 at Thr209, Thr387, and Ser376 residues, which triggers IRAK1 autophosphorylation within the N-terminal ProST region, its dissociation from MyD88, and subsequent formation of an intermediary signalosome complex at the plasma membrane [21,25,28,50].

Taken together, the results of the present study suggest that PD-linked PINK1 positively regulates the early events of IL-1β-induced signaling. These findings further imply that PINK1 can contribute to PD pathogenesis by affecting the accompanying inflammatory response.

## Conclusions

While inflammation largely contributes to the pathogenesis of PD, the functional link between PINK1 and PD-linked neuroinflammation remains poorly understood. We previously revealed that PINK1 stimulates the IL-1β response by positively regulating two components of IL-1β-mediated downstream signaling, TRAF6 and TAK1. In the present study, we investigated whether PINK1 additionally affects other upstream molecules in the IL-1β signaling pathway as well as the formation of the intermediate signalosome complex. Here we show that PINK1 stimulates IL-1β-induced signaling via suppression of Tollip inhibitory action, potentiation of Lys63-linked IRAK1 ubiquitination, and facilitation of an intermediate signalosome complex formation containing IRAK1, TRAF6, and TAK1. These results suggest that PINK1 effectively modulates several sequential steps of IL-1β signaling through direct binding to multiple upstream and downstream proteins in IL-1β-mediated signaling pathway and modulating their interactions. The present studytherefore additionally suggests that PINK1 may contribute to the pathogenesis of PD by affecting the accompanying inflammatory response.

## Abbreviations

DMEM: Dulbecco’s modified Eagle’s medium; ERK: Extracellular signal-regulated kinase; FBS: Fetal bovine serum; FCS: Fetal calf serum; FITC: Fluorescein isothiocyanate; HEK293: Human embryonic kidney 293; IKK: IκB kinase; IL: Interleukin; IL-1R: IL-1 receptor; IRAK1: IL-1 receptor-associated kinase 1; MEF: Mouse embryonic fibroblast; MyD88: Myeloid differentiation factor 88; NF: Nuclear factor; PBS: Phosphate-buffered saline; PD: Parkinson disease; PINK1: PTEN-induced putative kinase 1; TAK1: Transforming growth factor-β activated kinase1; TLR: Toll-like receptor; TNF: Tumor necrosis factor; Tollip: Toll-interacting protein; TRAF6: TNF receptor-associated factor 6; TRITC: Tetramethylrhodamineiso-thiocyanate.

## Competing interests

The authors declare that they have no competing interests.

## Authors’ contributions

HJL designed the study, carried out the whole experiments, analyzed data, and drafted the manuscript. KCC designed the study, analyzed data, and wrote manuscript. Both authors read and approved the final manuscript.
